# Glioblastoma Circulating Cells: Reality, Trap or Illusion?

**DOI:** 10.1155/2015/182985

**Published:** 2015-05-20

**Authors:** A. Lombard, N. Goffart, B. Rogister

**Affiliations:** ^1^Laboratory of Developmental Neurobiology, GIGA-Neuroscience, University of Liège, Liège, Belgium; ^2^Department of Neurosurgery, CHU and University of Liège, Liège, Belgium; ^3^Department of Neurology, CHU and University of Liège, Liège, Belgium; ^4^GIGA-Development, Stem Cells and Regenerative Medicine, University of Liège, Liège, Belgium

## Abstract

Metastases are the hallmark of cancer. This event is in direct relationship with the ability of cancer cells to leave the tumor mass and travel long distances within the bloodstream and/or lymphatic vessels. Glioblastoma multiforme (GBM), the most frequent primary brain neoplasm, is mainly characterized by a dismal prognosis. The usual fatal issue for GBM patients is a consequence of local recurrence that is observed most of the time without any distant metastases. However, it has recently been documented that GBM cells could be isolated from the bloodstream in several studies. This observation raises the question of the possible involvement of glioblastoma-circulating cells in GBM deadly recurrence by a “homing metastasis” process. Therefore, we think it is important to review the already known molecular mechanisms underlying circulating tumor cells (CTC) specific properties, emphasizing their epithelial to mesenchymal transition (EMT) abilities and their possible involvement in tumor initiation. The idea is here to review these mechanisms and speculate on how relevant they could be applied in the forthcoming battles against GBM.

## 1. Introduction

Circulating tumor cells (CTC) are the main required substrate for cancer to spread and extend metastases. These cells originally come from the primary tumor and reach the vascular compartment. CTC are then able to leave the circulation, migrate through the conjunctive tissue of different organs, and proliferate to form metastases. It remains unclear whether CTC are able to go back to the primary tumor site, specifically after therapeutic treatment, and therefore to participate to tumor recurrence.

In fact, it has been suggested that a very small proportion of CTC can form metastases. This subpopulation of cells is called circulating tumor stem cells (CTSC). Indeed, this subpopulation is thought to be self-renewing, multipotent, and capable of tumor initiation [1]. Up to now, different hypotheses try to explain their presence in the peripheral blood, involving several mechanisms to cross the vascular barrier. Because of their properties, these cells are of high interest to counteract the evolution of the disease and metastases formation. This review aims to better understand the biology of these CTSC with a particular focus on glioblastoma multiforme, a grade IV malignant brain tumor characterized by a dead-end prognosis, systematic relapses, and rare metastases.

## 2. Origins, Circulation, and Destinations of Circulating Tumor Stem Cells (CTSC)

CTC come from the initial tumor or from eventual metastases. In the tumor mass, less than 5% of malignant cells [2] are known to preserve a self-renewal potential through multiple generations and are able to create a new tumor. These are called cancer stem cells (CSC). Classically, CSC are defined by three major* in vitro* properties: formation of spherical colonies in culture suspension, differential levels and patterns of surface markers, and increased survival after radiation or chemotherapeutic treatment [3–7]. Moreover, in experimental models, those CSC are the only tumor cells able to initiate the development of new tumors in heterotopic or homotopic xenotransplantation experiments. These CSC present high tolerance to the lethal environment, host defense and growth-suppression factors thanks to immune mediators, cell cycle checkpoints, and DNA damage control pathways [8].

**Figure 1:**
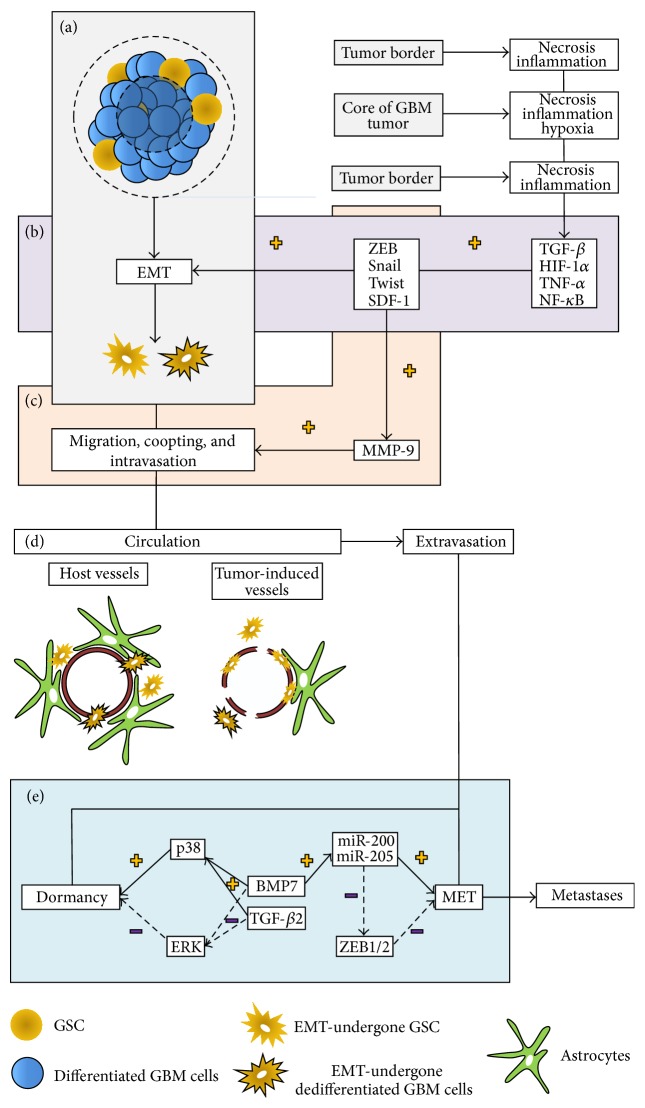
Insights on GBM dissemination process. Both GSC and differentiated cells can undergo EMT in order to invade the brain parenchyma. This process is regulated by different transcription factors including ZEB, SNAIL, Twist, or NF-*κ*B that are activated upon several environmental conditions (inflammation, necrosis, and hypoxia) ((a) and (b)). This consequently results in the acquisition of mesenchymal properties and the expression of ECM degrading enzymes in order to favor tumor spread. This process also sustains intravasation, leading to systemic dissemination ((c) and (d)). Tumor blood vessels are usually incomplete and leaky, therefore favoring intra-/extravasation (d). In pathological conditions, the BBB is often disrupted, facilitating GBM cells to jump in the blood flow as well (d). When tumor cells extravasate, they may either become quiescent or develop metastases. This balance is tightly regulated by environmental conditions and factors including BMP7 or TGF*β*2 among many others which may induce either dormancy or a switch toward MET and metastases (e).

From this, different hypotheses attempted to elucidate the presence of CSC in the blood or circulating tumor stem cells (CTSC). CSC can use a normal morphogenetic process called Epithelial Mesenchymal Transition (EMT) [9] to modify their features in order to escape the tissue of origin and to migrate towards the vascular compartment [10]. Liu and collaborators recently demonstrated that differentiated tumor cells acquire migratory abilities due to the development of EMT pathways [11] (Figure 1(a)). The intravasation is finally possible by the secretion of enzymes, such as serine/cysteine proteases, matrix metalloproteases (MMP) or disintegrins, and other metalloproteases (ADAMS), in order to degrade the basal membrane of blood vessels [12]. The presence of tumor-associated macrophages (TAMs), especially in hypoxic region of tumor [13], seems indeed to facilitate the intravasation process, maybe via secretion of MMP-9 [14].

Once in the bloodstream, most of the CTC, including CSC, undergo an important selection by shear forces or natural killer (NK) cells from the immune system [15]. However, CTC can aggregate to cellular elements [16] or platelets [17] and express several receptor tyrosine kinases (RTK), antiapoptotic molecules and invasion signaling components [16, 18]. CTC in this way are able to avoid not only the immune response but also anoikis [18]. To extravasate, CTC use diapedesis to escape the vascular compartment [19]. Then, CTC that present mesenchymal features can inverse their transition and then recover their epithelial phenotype of origin via a process called Mesenchymal to epithelial transition (MET). Some CTC finally become quiescent in a new and favorable environment and can later on fully participate to cancer relapses (Figure 1(e)).

## 3. EMT Conditions and Molecular Regulation

If CTC are the substrate, EMT might be a necessary condition for cancer dissemination. EMT is indeed thought to be the program that cancer cells follow to acquire metastatic features. This substantially simplifies our conception of the metastatic cascade, even if EMT is certainly not sufficient. EMT/MET is a normal embryologic reversible program that allows the conversion of epithelial cells to mesenchymal cells and inversely during development. Its embryonic implication, especially in gastrulation, neural crest delamination, and organ formation and development, is well described [20]. Later, in response to injuries, EMT was shown to be induced by EGF [21] and used by keratinocytes in healing process [22].

The corner stone of EMT/MET processes is the down-/upregulation of E-Cadherin (E-Cad), an integral membrane protein and a component of adherent junctions and an important mediator of cell-cell adhesion. The* CDH1* gene encodes E-Cad. It can be repressed in two ways, depending on the effect on the E-cadherin promoter. First, transcriptional repressors including Snail, Slug, ZEB1, and ZEB2 (zinc finger proteins) and basic helix-loop-helix (bHLH) such as E47 transcription factor bind directly to E-boxes of the* CDH1* promoter region [23–26]. Kruppel-like Factor 8 also represses E-Cad expression by fixing* CDH1* promoter in an E-box independent way [27]. Second, the bHLH Twist1 factor, E2-2 factors, and the embryonic transcription factor Goosecoid indirectly repress the* CDH1* transcription [28, 29]. Interestingly, Snail and Twist appear to control positively ZEB1 expression [30].

Many EMT inducers are currently known. Nuclear factor kappa-B (NF-*κ*B) has a putative binding site on the* Snail* promoter, inducing Snail protein and preventing its phosphorylation by glycogen synthase kinase-3 (GSK-3) and its subsequent degradation [31]. It has been shown that tumor necrosis factor *α* (TNF-*α*) induces and stabilizes Snail protein via NF-*κ*B [32]. Transforming growth factor-*β* (TGF-*β*) is a well-known EMT inducer. The binding of TGF-*β* to its receptor leads to phosphorylation of Smad transcription factors, which strongly induce Snail and Twist expression, particularly in presence of high-mobility group protein HMGA 2 [33]. Protein kinase A (PKA), signal transducer and activator of transcription 3 (STAT3), and protein kinase D (PKD) are involved in TGF-*β*-induced EMT [34, 35].

Local conditions could also modulate the EMT process. This is the case of hypoxia, a local condition frequently encountered in the tumor mass. Indeed, during hypoxia, Notch pathway is activated, resulting in Notch intracellular domain (NICD) liberation. NICD acts then as a transcription factor that interacts with DNA-binding protein CSL to regulate gene expression. NICD particularly upregulates Snail expression by direct binding to its promoter [36]. Similarly, still in hypoxic condition, hypoxia-inducible factor-1 (HIF-1), potentiated by Notch, is able to stabilize Snail by recruiting lysyl-oxidase (LOX) [36]. However, HIF-1 can also induce Twist expression by binding directly to the hypoxia-response element (HRE) to the* Twist* promoter sequence [37]. As another factor was upregulated during hypoxia or inflammation, vascular-endothelial growth factor or VEGF can induce Twist and Snail expression by GSK-3 inhibition [38, 39]. The same regulation of Twist and Snail expression is observed with EGF as it can particularly act in cooperation with *α*5*β*1 integrin [40]. Sonic-Hedgehog pathway is also related to Snail expression, probably induced by Gli1 [41] and contributes to TGF-*β*-induced EMT [42]. Hyperactive Wnt signaling occurs with the progression of different carcinomas and it has been shown that Wnt stabilizes Snail (and therefore EMT) by GSK-3B inhibition via Axin-2 [43]. Thus, EMT appears to be the result of E-Cad repressors activities, especially Snail factors, in response to inflammation and hypoxic conditions [44], both features that are met in cancer.

On the other side, another pathway including miRNAs is well known for its rule in epithelial transition. Bone morphogenetic protein (BMP) pathway, especially via BMP7, induces miR-205 and miR-200 family of microRNAs, which induce* CDH1* promoter and suppress ZEB1 and ZEB2 expression [45], and thus promotes MET [46] (Figure 1(e)).

## 4. EMT-Related Changes

During EMT, epithelial cancer cells, which lean bit by bit towards the mesenchymal state, loose epithelial features and change their protein expression. Hence, it is possible to characterize the CTC epithelial or mesenchymal phenotype and specifically the degree of transition. Epithelial cellular adhesion molecule (EpCAM) [47], cytokeratins [48],* zonula occludens* [49], or epithelial splicing regulator 1 (ESPR1) [50] expression characterize an epithelial phenotype, while N-Cadherin [51] or Vimentin [52] are expressed in mesenchymal phenotype. Activation of biochemical pathways, such as Twist-1 or the Akt-PI3K pathway [53], can also be specific hallmarks of the mesenchymal state. EMT is associated with the acquisition of several properties that are critical for cancer dissemination including first repression of the epithelial cell polarity and proliferation, and second, promotion of cell resistance to therapy, migration, and invasion [20]. Inversely, MET promotes cell proliferation and metastasis formation.

E-Cad repressors as well as EMT inducers are involved in this acquisition. For example, Snail factors induce MMP-9 expression that is then able to degrade the basement membrane of blood vessels, a prerequisite step to intravasation. Conversely, some metalloproteases, such as MMP-3 and MMP-13, can induce EMT [54, 55]. Additionally, TGF-*β* confers resistance to cell death and DNA damage [56]. In fact, Snail and Slug factors repress proapoptotic genes expression, in particular PUMA, ATM, and PTEN that are usually upregulated in the p53-mediated apoptotic pathway [57]. In the same line, Twist1 and Twist2 were shown to be overexpressed in a large fraction of human cancers and are thus able to override the oncogene-induced premature senescence by abrogating key regulators of the p53- and Rb-dependent pathways. In epithelial cells, the oncogenic cooperation between Twist proteins and activated mitogenic oncoproteins led to complete EMT. Taken together, these data underlined an unexpected link between early escape from failsafe programs and the acquisition of invasive features by cancer cells [58]. EMT is also associated with chemo- and radio-resistance. Snail indeed inactivates p53-mediated apoptosis [57], whereas Twist upregulates the serine/threonine kinase AKT2 [59]. Finally, Kudo-Saito et al. showed in melanoma that Snail positive tumor cells have recourse to thrombospondin-1 (TSP-1) in order to impair dendritic cells, resulting in CD4+ regulatory T cells induction with immunosuppressive capacity, hence promoting immunoresistance, immunosuppression, and/or escape of immune surveillance [60].

Mesenchymal transition also appears to confer or enhance stem cell properties by activation of Ras/MAPK pathway [61] (Figures 1(a) and 1(b)). Snail factors can indeed promote the Wnt pathway (known for its regulation in self-renewal and differentiation in stem cells) by E-Cad repression [62]. ZEB1 and ZEB2 factors downregulate some specific members of the microRNAs 200 family (miR-200), particularly miR-200c, which targets the polycomb group member BMI1, an essential regulator of stem-cell renewal, acting as a repressor of various genes by modulating the chromatin status [45, 63, 64]. More and more reports highlight the importance of the miR-200/ZEB feedback loop in determining epithelial and mesenchymal future of tumor cells [64]. In the same way, Lu et al. used the loop to define three different states in the continuum between the epithelial and mesenchymal differentiation: epithelial (high miR-200/low ZEB), mesenchymal (low miR-200/high ZEB), and partial EMT (medium miR-200/medium ZEB) [65]. The acquisition of stem cell properties could explain the possible various origins of CSC.

No matter the epithelial or mesenchymal state, some cell markers suggest stemness character in CTC. In breast cancer for instance, aldehyde dehydrogenase-1 (ALDH1) allows to detect CTC with CSC properties [53]. The expression of cell surface markers CD44+/CD24− is also associated with CSC in breast carcinoma and with CTSC in colon carcinomas [66]. Gangliosides (GD2, GD3, and GD1A) in breast cancer and ABC proteins (ABCG2) in lung cancer are also useful for stemness detection [67, 68], but their utility for CTSC detection remains uncertain.

## 5. Dormancy

Tumor cells that are physically separated from the primary tumor mass and have spread to other anatomical locations through circulation are called disseminated tumor cells (DTC). They can be classified as a subgroup of CTC. Metastasis formation is one option that DTC can follow but some of them are also able to become quiescent, a process that is different from senescence and consists in a nonproliferative state consequent to cell cycle arrest in phase G0/G1 [69]. Quiescence results from mitogenic signaling reduction and implies autophagy [70], reduced PI3K-AKT signaling [71], and activation of stress signaling pathways [72]. Interestingly, dormancy is significantly influenced by the microenvironment which can be permissive or restrictive [73]. In the bone marrow compartment, the presence of proteins such as GAS6, BMP4, BMP7, and TGF-*β*2 confers an adequate environment for dormancy [74–76], whereas VCAM1, periostin, and extracellular matrix stiffness, with high density of type I collagen, appear to induce escape of dormancy [77–79]. Many key players modulate tumor cells dormancy. Among them, the balance of two prominent pathways, p38 mitogen-activated protein kinase (MAPK) and extracellular signal-regulated kinase (ERK), might be key determining factors [75]. A high ratio of ERK/p38 is observed in metastatic lesions [80], while low ratio of ERK/p38 is associated with dormancy [81]. In addition, inactivation of Myc oncogene also leads to senescence [82].

Multiple actors are involved in quiescence process. For example, fibroblasts express periostin, which recruits Wnt pathway ligands and increases Wnt signaling in cancer stem cells, resulting in metastatic colonization [83]. In the bone marrow stem cell niche, stromal cells, such as osteoblasts, via TGF-*β*2, induce low radio of ERK/p-38 and p27 expression, inhibit CDK4, and in this way induce cancer cell quiescence [81]. In the same way, bone morphogenetic protein 7 (BMP-7) binds to BMPR2 that activates p38 and increases the expression of cell cycle inhibitor p21 and metastasis suppressor gene NDRG1 (N-myc downstream-regulated Gene 1) [75]. Macrophages, CD4+ and CD8+ T cells, founded in immune niche, use tumor necrosis factor receptor 1 (TNFR1) and interferon-*γ* (IFN-*γ*) in order to induce antiangiogenic chemokines and prevent proliferation and carcinogenesis [84]. More specifically, CD4+ T cells products CXCL9 and CXCL10, which were described, inhibit angiogenesis [85]. Endothelial cells from bone marrow vascular niches can also induce quiescence via TSP-1 or perlecan production [78, 86].

Dormancy appears as an important phenomenon in the cancer relapse as it implies higher resistance against targeted and conventional therapies, and after long period, sometimes decades, tumor cells can quit this specific dormancy state and develop regrowth capacities [87]. A strong link between dormancy state and tumor stem cells is suspected [88]. Indeed, both dormant tumor cells and tumor stem cells show a high resistance to current treatments [89, 90] and can undergo cell cycle arrest in response to different form of therapy [91, 92]. In glioblastoma, for example, the CSC pool in tumors is enriched after ionizing radiation. This situation seems to be in direct consequence with the activation of DNA damage repair pathways coupled to a reduction of proliferation and apoptosis via DNA checkpoint kinases [93]. In fact, a subpopulation of CSC is thought to be quiescent [94]. This view is supported by the fact that dormant cells and CSC use the same pathways such as Shh, Notch, and Wnt [95]. The overlap between dormancy state and ability of tumor-initiation could help to determinate the subpopulation of tumor cells, which are highly involved in relapses.

## 6. CTSC and Glioblastoma

### 6.1. Clinical Evidence

GBM is the most frequent primary brain tumor and is well known for its poor prognosis despite multimodal therapies. The rapid relapse of tumor in GBM patients has indeed been regarded for years as the major cause of the lack of GBM spread out of the central nervous system. However, there are several clinical descriptions of glioblastoma metastasis. In 1928, Davis and colleagues described the first case ever reported of glioblastoma metastasis in a 31-year-old woman. Since then, a growing body of evidence has shown the capacity of GBM to spread not only via the cerebrospinal fluid (CSF) but also via blood or lymphatic vessels [96, 97] (Figure 1(e)). Interestingly, the number of GBM metastatic reports increases progressively [98]. This could be explained by a higher rate of diagnosis not only due to imaging improvement but also due to the modest but real increase of patient survival and outcomes. Interestingly, the incidence of glioma metastases on postmortem examinations ranges from 6 to 25% for supratentorial tumors [99, 100]. The actual delay between the initial tumor diagnosis and metastases found in the literature is 1 to 60 months [101]. Thus, clinical evidences allow to asserting the existence of CTC and DTC in GBM.

### 6.2. CSC in Glioblastoma

Ignatova et al. first highlighted the presence of CSC in GBM [102] (Figure 1(a)). Many similarities exist between GSC and normal stem cells in the adult brain, also termed neural stem cells (NSC). These populations indeed share particular resemblances in gene expression and signaling pathways including Notch, Wnt, or TGF-*β* signaling [103–105]. CD133 or prominin-1 was proposed as a biomarker of tumor progression/initiation cells described in glioblastoma [106], but it appeared later to be insufficient as CD133-negative cells were also able to initiate tumors [107]. Interestingly, not only Sox2 (a transcription factor) but also nestin (an intermediate filament protein) and integrin *α*6 expression are highly expressed in GSC population [108, 109]. EGFR, whose amplification and mutations are well known in GBM, also promotes stemness in GBM cells [110]. Although it is unclear whether GSC result from cancerous transformation of NSC, they have been demonstrated to preferentially locate in specific niches, more specifically in neurogenic niches, such as subventricular zone [111]. Evidence also considers their presence in necrotic niches [112] or in tumor edge niches [113].

### 6.3. Defective Brain-Blood Barrier (BBB) and GBM-Circulating Cells

The blood brain barrier (BBB) consists basically of endothelial cells connected by tight junctions, surrounded by astrocytic endfeet with pericytes embedded in the vessel basal membrane. Nevertheless, neurons and microglia are also implicated in the BBB cytoarchitecture [114]. In fact, a double interaction exists between endothelial cells and astrocytes, called gliovascular coupling. While endothelial cell can stimulate astrocytic growth and differentiation, astrocytes also modulate tight junctions formation and angiogenesis via the src-suppressed C-kinase substrate (SSeCKS) [115, 116]. Moreover, astrocytic endfeet use aquaporins (AQP) to maintain the BBB integrity [117].

GBM is the most vascularized tumor in humans [118]. Among others, this can be explained by high levels of vascular endothelial growth factor (VEGF), particularly in necrotic core, resulting in endothelial proliferation [119]. Nevertheless, glioblastoma-induced angiogenesis is imperfect leading to vessel formation with variable diameter and permeability, heterogeneous distribution, and basal lamina irregularities [120] (Figures 1(c) and 1(d)). In 1975, Hirano and Matsui had already shown fenestrations and tight junctions disruption in GBM vessels [121]. At the beginning, GBM cells use host vessels as pathways of invasion [122] and, then, co-opt to these vessels [123]. These interactions of GBM cells with vessels become more and more prominent as the disease progresses. Indeed, new-generated vessels by angiogenesis can support tumor growth, with a tone controlled by glioma cells [124]. Watkins et al. also showed that glioma cells displace, or even eliminate, astrocytic endfeet and make direct contacts with endothelial cells (Figures 1(c) and 1(d)). The result is, first, the cessation of endothelial/astrocytic interaction and, second, the breach of BBB, by reduction of tight junctions [124]. Thus, glioblastoma progression seems to tightly associate with altered BBB permeability, which also constitutes the first condition to intravasate.

### 6.4. Glioblastoma Subtypes and EMT: The Mesenchymal Link

Based on gene expression signatures, four GBM subtypes have been described: proneural, neural, classical, and mesenchymal [125]. In particular, the mesenchymal subtype is characterized by high expression of CHI3L1 and MET, wild-type IDH1, mutation/deletion of* NF1*, Schwann-like features, and important presence of necrosis/inflammation [125–127]. This subtype is usually associated with worse prognosis and most of the time, appears* de novo* [128, 129]. Fibronectin and collagen 5*α*1 are used as markers of mesenchymal GBM subtype [125]. Some regulators of mesenchymal status have also been highlighted in this subtype, such as C/EBP-B and STAT3 transcription factors or the transcriptional coactivator TAZ [130, 131].

In this context, Bhat and colleagues have recently shown that microglia are able to induce the mesenchymal status via the TNF-*α*/NF-*κ*B pathway, notably resulting in radioresistance [132]. Moreover, it has been shown that the mesenchymal phenotype is associated with higher migratory capacities of GBM cells. In fact, TGF-*β*, which is well present in the GBM environment and secreted by microglia, stromal and tumor cells [133], is able to induce the mesenchymal transition, via SMAD2 phosphorylation and recruitment of ZEB1, especially in GBM with a low or absent expression of mesenchymal markers [134]. This mesenchymal differentiation can be effectively blocked by A8301, an inhibitor of the TGF-*β* type 1 receptors [134]. Hypoxia, via HIF-1*α* and ZEB1, is also able to induce a mesenchymal switch in GBM [135]. Moreover, Twist overexpression enhances GBM invasion [136]. Snail is also upregulated in glioma cells compared to normal brain cells and was shown to promote invasion [137]. Indeed, its inactivation inhibits glioma progression and migration [138]. Finally, stromal cell-derived factor (SDF-1) or CXCL12 and its receptor CXCR4 can induce EMT in GBM via activation PI3K/Akt and ERK pathways [139]. Interestingly another recent study reported the involvement of the CXCL12/CXCR4 axis in EMT transition via upregulation of survivin, a protein involved in apoptosis inhibition [140]. Moreover survivin-mediated EMT was shown to promote resistance to *γ*-radiation, suggesting a potential role of EMT in GBM therapeutic resistance [141].

As mesenchymal transition is associated with the acquisition of stem cell properties, hypoxia seems to increase stem-cell markers in GBM cells, via HIF-1*α* and Notch inductions [142]. Speaking about new properties, mesenchymal transition in GBM was shown to confer tumor resistance to anti-VEGF therapy [143].

GBM metastases are not* stricto sensu* associated with the mesenchymal subtype. This is no surprise as it has been demonstrated that different subtypes of GBM can coexist within the same tumor [144]. Moreover, Ozawa et al. showed that GBM could derive from a common proneural-like precursor and that additional NF1 loss can convert this proneural subtype to a mesenchymal subtype [145]. Thus, mesenchymal transition can be understood as a late phenomenon in GBM, leading to more aggressive, invasive, and recurrent tumor. This idea is supported by the fact that mesenchymal subtype is frequently found in glioblastoma metastases and recurrences [146].

### 6.5. CTSC in Glioblastoma

Recently, CTC have been found in GBM patients' blood, highlighted by GFAP detection, EGFR amplification, or increased telomerase activity [147, 148]. The phenotypes of these CTC in GBM patients were closed to the mesenchymal or proneural subtypes. However, recent studies have not found stemness features in those cells yet, but it does not rule out that some of these CTC are also indeed true CTSC. Of course, this hypothesis is sustained by clinical evidences and the existence of GBM metastases [98]. Recently, Song et al. showed that MMP-9 is required to cross the BBB, especially the parenchymal barrier [149]. Interestingly, as Snail also induces MMP-9 expression, the mesenchymal transition therefore seems a necessary condition to intravasate (Figure 1(c)). Besides, as dormancy is also a reality in GBM [150], we can speculate that some of these CTC remain quiescent in other tissue and could later on initiate relapses. Moreover, circulating endothelial cells and circulating hematopoietic progenitor cells also appear to be present in GBM [151]. Interestingly, GSC have the ability to differentiate into endothelial cells and show the ability to generate new tumors when grafted in immunodeficient mice [152] (Figure 1(d)). This reinforces even more the hypothesis according to which CTSC are an underrated reality in GBM.

## 7. Conclusion

For many years, GBM was thought to be restricted to the central nervous system but a growing body of evidence indicates that, like many other cancers, hematogenic dissemination is a reality. CTC characterization is needed to confirm the presence of CTSC. The question of a possible CTC role in GBM relapses remains open. We think this is a crucial question to address as its response could significantly modify actual therapeutic protocols and have an important impact on patient outcomes.
